# Identification of Finely Mapped Quantitative Trait Locus and Candidate Gene Mining for the Three-Seeded Pod Trait in Soybean

**DOI:** 10.3389/fpls.2021.715488

**Published:** 2021-11-26

**Authors:** Candong Li, Hongwei Jiang, Yingying Li, Chunyan Liu, Zhaoming Qi, Xiaoxia Wu, Zhanguo Zhang, Zhenbang Hu, Rongsheng Zhu, Tai Guo, Zhixin Wang, Wei Zheng, Zhenyu Zhang, Haihong Zhao, Nannan Wang, Dapeng Shan, Dawei Xin, Feishi Luan, Qingshan Chen

**Affiliations:** ^1^Jiamusi Branch Institute, Heilongjiang Academy of Agricultural Sciences, Jiamusi, China; ^2^College of Life Science, Northeast Agricultural University, Harbin, China; ^3^Country College of Agriculture, Northeast Agricultural University, Harbin, China; ^4^Soybean Research Institute, Jilin Academy of Agricultural Sciences, Changchun, China; ^5^Key Laboratory of Molecular Epigenetics of MOE, Institute of Genetics and Cytology, Northeast Normal University, Changchun, China

**Keywords:** soybean, three seeded pods, QTL fine mapping, candidate genes analysis, gene function identification

## Abstract

The three-seeded pod number is an important trait that positively influences soybean yield. Soybean variety with increased three-seeded pod number contributes to the seed number/plant and higher yield. The candidate genes of the three-seeded pod may be the key for improving soybean yield. In this study, identification and validation of candidate genes for three-seeded pod has been carried out. First, a total of 36 quantitative trait locus (QTL) were detected from the investigation of recombinant inbred lines including 147 individuals derived from a cross between Charleston and Dongning 594 cultivars. Five consensus QTLs were integrated. Second, an introgressed line CSSL-182 carrying the target segment for the trait from the donor parent was selected to verify the consensus QTL based on its phenotype. Third, a secondary group was constructed by backcrossing with CSSL-182, and two QTLs were confirmed. There were a total of 162 genes in the two QTLs. The mining of candidate genes resulted in the annotation of eight genes with functions related to pod and seed sets. Finally, haplotype analysis and quantitative reverse transcriptase real-time PCR were carried to verify the candidate genes. Four of these genes had different haplotypes in the resource group, and the differences in the phenotype were highly significant. Moreover, the differences in the expression of the four genes during pod and seed development were also significant. These four genes were probably related to the development process underlying the three-seeded pod in soybean. Herein, we discuss the past and present studies related to the three-seeded pod trait in soybean.

## Introduction

Traits related to pods and seeds are key factors influencing soybean (*Glycine max* L. Merrill) yield. The soybean yield is determined by evaluating direct yield-related traits at a set planting density, including seed number per plant and 100-seed weight. Seed number per plant, the parameter most related to yield potential (Bárbaro et al., 2006), is closely associated with the number of pods per plant and with seeds per pod. The seeded-pod trait is a quantitative trait that is highly susceptible to environmental conditions, which leads to ovule abortion and the emergence of various seeded pods on a single plant. This trait also has a certain genetic heritability in different varieties. An increase in the number of three- or four-seeded pods theoretically increases soybean yield. However, a previous study revealed a strong negative correlation between the number of four-seeded pods and 100-seed weight (Carvalho et al., 2017). Another recent study indicated that an increase in the number of four-seeded pods caused decreased seed size, resulting in no yield increase (Li et al., 2018). In soybean production, compared with other varieties, those plants with many three-seeded pods may possess more pods and larger seeds (Zhou et al., 2005; Tavares et al., 2013). The number of three-seeded pods per plant (NThSP) is an important trait for soybean breeding. The “Henong 71” cultivar, which has more three-seeded pods than other varieties (the proportions of two- and three-seeded pods are 10.70 and 82.68%, respectively), produced a record high yield for the Xinjiang Production and Construction Corps of China in 2019. The test yield was 6,712 kg/ha (Guo et al., 2020).

The NThSP is a quantitative trait that is easily affected by the environment. It is difficult to accurately select this trait during breeding. Marker-assisted selection and breeding by design can solve this problem *via* the use of functional molecular markers and genes. Thus, candidate gene mining and subsequent functional verification of genes affecting NThSP are important. Recent advances have been made in quantitative trait locus (QTL) mapping in specific populations, including the gradual improvements of statistical methods and the continual development of genetic maps (Cregan et al., 1999; Song et al., 2004; Choi et al., 2007; Hyten et al., 2010), the gradual enhancement of the technology used for phenotypic analyses of soybean, and increases in the throughput and affordability of genome resequencing. These changes have benefited research aimed at identifying QTL related to NThSP. For example, more than 120 NThSP QTLs are distributed on 20 chromosomes in soybean^1^^,2^. These QTLs were detected during investigations of other soybean yield-related traits. To date, NThSP QTLs have not been comprehensively identified or analyzed.

Recently, NThSP QTLs were detected and mapped primarily in recombinant inbred lines (RILs). Several RILs were derived from various hybridizations. First, 15 NThSP QTLs were detected in RILs resulting from the cross between “Jin 23” and “Huibuzhiheidou” (with high three-seeded pod yield). The mean phenotypic variance explained (MPVE) by these QTLs was 1.5–34.70%. Second, an analysis of the RILs derived from the “Zhongdou 29” × “Zhongdou 32” (with high three-seeded pod yield) hybridization detected 13 NThSP QTLs, with an MPVE of 2.54–57.54%. Third, 47 NThSP QTLs were detected in RILs derived from a cross between “Charleston” and “DongNong594” (with a high three-seeded pod yield), with an MPVE of 0.70–46.70%. Finally, two RILs resulting from the “Heihe 36” × “DongnongL 13” (with high three-seeded pod yield) and “Dongnong L13” (with a high three-seeded pod yield) × “Henong 60” hybridizations were analyzed, resulting in the detection of 20 NThSP QTLs, with an MPVE of 0.71–11.79%. Additionally, four-way RILs were evaluated to identify NThSP QTL (Supplementary Table 1).

Although many NThSP QTLs have been identified, their genomic locations are often undetermined. Moreover, there have been relatively few studies on the candidate gene mining and functional verification of NThSP QTL in soybean. In this study, NThSP QTLs were detected in a RIL population. Meta-QTLs (MQTLs) were subjected to a meta-analysis. The MQTLs were verified using a wild soybean whole-genome introgression line. A secondary group with the target interval was constructed, and the bulked segregant analysis (BSA) resequencing technology was applied for QTL fine mapping and candidate gene mining.

## Materials and Methods

### Plant Materials and Experimental Design

For QTL identification, a RIL comprising 147 individuals was obtained from a cross between an American soybean cultivar (“Charleston” NThSP 20.01) as the female parent (Cooper et al., 1995) and a Chinese cultivar (“DongNong594” NThSP 26.40) as the male parent, which was developed at Northeast Agricultural University, Harbin, Heilongjiang, China. Single seed descent was used to produce F_2:20_–F_2:23_ generations (Chen et al., 2005). For *MQTL* validation and secondary group construction, a chromosomal segment substitution line (CSSL) consisting of 220 individuals from the BC_3_F_3_, BC_3_F_4_, BC_3_F_5_, and BC_3_F_6_ generations was backcrossed with the recurrent parent, “Suinong 14” (developed at Suihua Branch Academy of Heilongjiang Academy of Agricultural Sciences, Suihua, Heilongjiang, China, NThSP 21.87), and the donor parent, “ZYD00006” (wild soybean from the China Germplasm Bank, NThSP 133.20). The linkage group was constructed as described by Xin et al. (2016). Each line in both populations and their parents were grown in Harbin, China (45.75°N, 126.53°E) from 2013 to 2016, according to a randomized complete block design with three replicates. In the experimental plot, the rows were 5 m long, with each row separated by 0.65 m and a plant spacing of 0.05 m.

### Data Collection and Quantitative Trait Locus Identification

The traits related to the seed number of pods containing one, two, three, four, and total seeds were recorded as NOSP, NTSP, NThSP, NFSP, and NPPP, respectively. The ratio of the three-seeded pods was determined as the percentage of NThSP to NPPP (Ning et al., 2018). The phenotype data for RILs and their parents were recorded in an earlier study by Li et al., 2018. The average data for five plants per line, randomly selected in each row, were calculated. The QTLs were detected using the composite interval mapping model of the QTL Cartographer (version 2.5) (Wang et al., 2006). The thresholds for detecting QTL were a minimum logarithm of odds score of 2.5 and *P* = 0.05 and were determined using 1,000 permutations at 1-cM intervals. The meta-analysis was performed using tools-meta analysis of the BioMercator 2.1 program. Consensus QTLs were detected according to the optimized model with the lowest Akaike information criterion values. The QTLs were named as previously described (McCouch et al., 1997) (i.e., q + trait name + linkage group number + “-” + QTL number).

### Consensus Quantitative Trait Locus Validation and Secondary Group Construction

Targeted lines carrying segments with consensus QTL physical intervals were selected from the 220 CSSLs. The NThSP phenotypic variance between the targeted lines and the recurrent parent was analyzed to validate the consensus QTL. After marker-assisted selection for validating the consensus QTL in the targeted lines, backcrosses with “Suinong 14” (recurrent parent), and two rounds of selfing, we obtained a secondary group comprising 970 BC_4_F_2_ individuals.

### Bulked Segregant Analysis and Fine Mapping

The BSA was used to anchor the candidate intervals (Xia et al., 2010; Song et al., 2016). Two bulks consisting of individuals with extreme phenotypes were selected from the secondary group. The CSSL-182 phenotype of the three-seeded pod trait was 39.22 and that of “Suinong 14” was 21.87. More specifically, 20 plants with more than 34 three-seeded pods (at least 70% of all pods) formed one bulk, whereas the other bulk consisted of 20 plants with fewer than 10 three-seeded pods (less than 30% of all pods). High-quality DNA was extracted from the parental lines and both bulks and used to construct deep-sequencing libraries for BSA. To obtain confident BSA analysis data, specific-locus amplified fragment sequencing (SLAF-seq) was employed, and the sequencing depth was 20 × for the selected soybean materials. Candidate intervals were confirmed using the single nucleotide polymorphism (SNP) index association mapping. The physical positions were determined according to the soybean reference genome (version 1.1) (Schmutz et al., 2010).

### Candidate Gene Mining

Candidate genes within the finely mapped QTL regions were predicted as previously described (Li et al., 2018). Interval marker sequences were obtained from the SoyBase database (see text footnote 1). Publicly available resources, including the Kyoto Encyclopedia of Genes and Genomes^3^ and the Gene Ontology^4^ databases, were used to detect and annotate candidate genes.

### Haplotype Analysis

Candidate gene genomic sequences were extracted from the Phytozome database^5^, including the promoter region upstream of the 5′ untranslated region (UTR), coding sequence (CDS), introns, and 3′ UTR. The basic local alignment search tool (BLAST) algorithm was used to compare the candidate gene genomic sequences with the whole-genome sequence of 92 germplasm resources (10 × sequencing depth), which were grown and phenotypically characterized as RILs and CSSLs. Germplasm resources included the main cultivars in Heilongjiang province, which had abundant phenotypic variation in seeded-pod-related traits. The number of seeded pods was 7.89–38.44, and the ratio of three-seeded pods was 30.99–61.22 (Supplementary Table 2). Haploview 4.2^6^ and Dnasp5.0^7^ were used to analyze the distribution of the main haplotypes in the germplasm resources. The 92 germplasm resources were divided into different classes according to their candidate gene haplotypes. The significance of the phenotypic differences among the haplotype groups of the germplasm resources was determined by ANOVA using the SPSS 20.0 program (IBM Corp., Armonk, NY, United States).

### Quantitative Real-Time PCR

**FIGURE 1:**
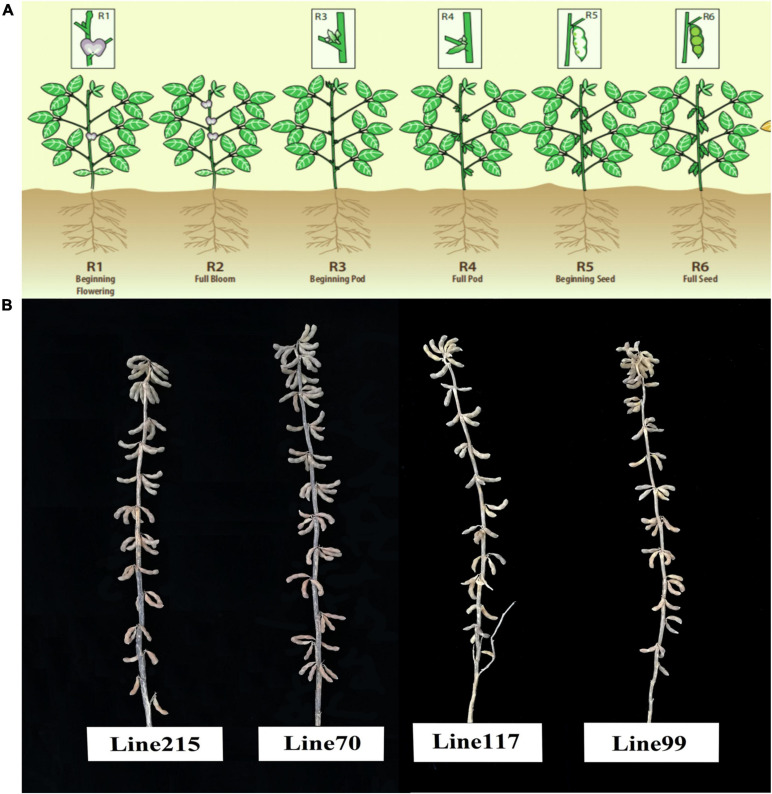
Sampling stages for the quantitative real-time PCR (qRT-PCR) and the individuals with an extreme phenotype regarding the number of three-seeded pods. **(A)** Sampling stages for the qRT-PCR. **(B)** Individuals with an extreme phenotype regarding the number of three-seeded pods. Lines 215 and 70 had a high number of three-seeded pods, whereas lines 117 and 99 had a low number of three-seeded pods.

**FIGURE 2:**
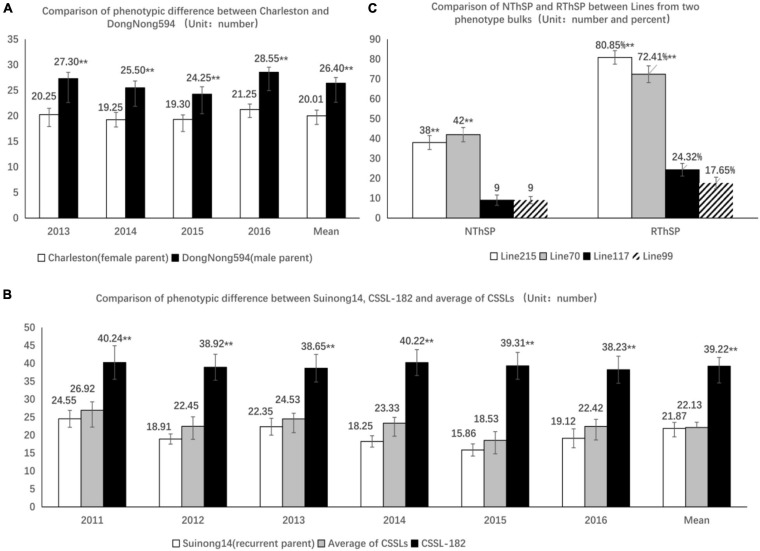
Differences in the number of three-seeded pods among different soybean lines. **(A)** Phenotype differences of the number of three-seeded pods between the parents of the recombinant inbred lines (RILs) (“Charleston” and “DongNong594”). The ordinate axis is the number of three-seeded pods. The horizontal axis is the year. **Significant difference (*P* < 0.01). **(B)** Phenotypic differences among CSSL-182 (i.e., chromosomal segment substitution line), the average chromosomal segment substitution line (CSSL), and the recurrent parent (Suinong 14). The ordinate axis is the number of three-seeded pods. The horizontal axis is the year. **Significant difference (*P* < 0.01). **(C)** Comparison of NThSP and RThSP in the lines from two phenotype bulks related to the number of three-seeded pods; NThSP represents the number of three-seeded pods, whereas RThSP represents the proportion of three-seeded pods. Lines 215 and 70 have high NThSP and RThSP values, whereas lines 117 and 99 have low NThSP and RThSP values. **Significant difference (*P* < 0.01). The ordinate axis is the number of three-seeded pods and the proportion of three-seeded pods. The horizontal axis is the number of three-seeded pods and the proportion of three-seeded pods.

The following four materials were selected from the secondary group: lines 215 and 70, which exhibited the high-NThSP-associated trait, and lines 117 and 99, which exhibited the low-NThSP-associated trait (Figure 1B). There were significant phenotypic differences between the two types of materials (*P* = 0.05) (Figure 2C). Flowers, pods, and seeds were collected at the following six stages: beginning flowering (R1), full flowering (R2), beginning pod (R3), full pod (R4), beginning seed (R5), and full seed (R6) (Figure 1A). The collected samples were stored at –80°C for subsequent RNA isolation. All samples were collected as three biological replicates, with each comprising three individual plants. The biological replicates were analyzed in triplicate. The *GmActin4* gene (GenBank ID: AF049106) was used as an internal control. The total RNA was extracted, cDNA was synthesized, and quantitative real-time PCR (qRT-PCR) analysis was performed as previously described (Jiang et al., 2018). The significance of the differences in candidate gene expression levels among the examined stages was determined using SPSS 20.0.

## Results

### Quantitative Trait Locus Detection and Integration

The NThSP data for the RILs were analyzed in an earlier study (Li et al., 2018). From 2013 to 2016, the average NThSP of “Charleston” was 20.01 and that of “DongNong594” was 26.40. The average NThSP of “Charleston” was 6.39 lower than that of “DongNong594.” The difference in NThSP between the two parents was significant (*P* < 0.01). The NThSP of the RILs varied significantly over the 4-year study period, ranging from 10.48 to 51.19 (Table 1 of Li et al., 2018 and Figure 2A).

**TABLE 1:** The consensus quantitative trait locus (QTL) integration of the number of three-seeded pods.

Consensus QTL	Chromosome	Genetic interval (cM)	Physical interval (Mbp)	QTL numbers	ADD^a^ range	Mean PVE^b^ (%)
*MQTL1*	GM02	52.5 ∼ 54.4	42.89 ∼ 46.19	5	−7.02 ∼ −2.36	15.74
*MQTL2*	GM03	1.90 ∼ 4.80	2.44 ∼ 4.63	4	−4.35 ∼ −2.25	7.01
*MQTL3*	GM12	26.5 ∼ 29.2	10.02 ∼ 11.20	4	−2.47 ∼ −0.17	3.01
*MQTL4*	GM13	9.3 ∼ 10.3	20.83 ∼ 26.06	4	−2.45 ∼ −1.42	2.97
*MQTL5*	GM17	90.1 ∼ 108.7	3.49 ∼ 6.72	5	−5.12 ∼ −2.56	10.07

*^a^ADD was the additive effects contributed by QTL.*

*^b^PVE was the phenotypic variation explained by QTL.*

A total of 36 NThSP QTLs were detected, which were distributed on GM02, 03, 12, 13, 17, 18, and 19. Of note, 16 QTLs had been published in a previous study (Li et al., 2018), and another 20 QTLs were detected in this study. At least four QTLs at the same location were selected for integration into a consensus QTL. In this study, five *MQTL* overlapped on GM02, 03, 12, 13, and 17 (Supplementary Tables 1, 3), and five NThSP QTL were integrated into *MQTL1*, with a genetic interval of 52.5–54.4 cM and a physical interval of 42.89–46.19 Mb. The additive effects ranged from –7.02 to –2.36, and the MPVE was 15.74%. Four NThSP QTLs were included in *MQTL2*, with a genetic interval of 1.90–4.80 cM and a physical interval of 2.44–4.63 Mb. The additive effects ranged from –4.35 to –2.25, and the MPVE was 7.01%. Both *MQTL3* and *MQTL4* comprised four NThSP QTLs, with negative additive effects and an MPVE of 3.01 and 2.97%, respectively. Five NThSP QTLs were included in *MQTL5*, with a genetic interval of 90.1–108.7 cM and a physical interval of 3.49–6.72 Mb. The additive effects ranged from –5.12 to –2.56, and the MPVE was 10.07% (Table 1).

### *MQTL* Validation

In the CSSL population, there were five lines containing donor segments that overlapped with *MQTL1*, CSSL-66, CSSL-75, CSSL-77, CSSL-158, and CSSL-203. Six lines contained donor segments that overlapped with *MQTL2*, CSSL-55, CSSL-123, CSSL-158, CSSL-171, CSSL-182, and CSSL-214. Six lines contained donor segments that overlapped with *MQTL3*, CSSL-46, CSSL-86, CSSL-103, CSSL-151, CSSL-157, and CSSL-182. Five lines contained donor segments that overlapped with *MQTL4*, CSSL-86, CSSL-103, CSSL-105, CSSL-118, and CSSL-182. Five lines contained donor segments that overlapped with *MQTL5*, CSSL-66, CSSL-75, CSSL-103, CSSL-158, and CSSL-182. An analysis of the NThSP data for these CSSL lines and the recurrent parent (Suinong 14) recorded from 2013 to 2016 indicated that the CSSL lines had higher NThSP than “Suinong 14.” The differences were extremely significant over the 4-year study period (Supplementary Table 4). This implies that the excellent phenotype of the CSSL lines was the result of the introgression of donor segments that overlapped with the *MQTL*. The *MQTLs* were validated using high generation genome-wide backcrossing introgression lines based on the phenotype. In particular, CSSL-182 is an excellent material containing donor segments that overlap with four *MQTLs*, namely, *MQTL2*, *MQTL3*, *MQTL4*, and *MQTL5*. The NThSP was significantly higher than in “Suinong 14” as well as the average NThSP for the CSSLs (Figure 2B).

Molecular marker analysis of CSSL-182 revealed 8 donor introgression segments, with 319 donor blocks (84.63 Mb) accounting for 8.93% of the whole genome. Moreover, 91.07% of the recurrent parent genome was restored by CSSL-182. The donor introgression segments of CSSL-182 included regions that overlapped with the *MQTL.* On GM03, a 2.19-Mb region overlapped *MQTL2* and the donor block. On GM12, a 1.18-Mb region overlapped *MQTL3* and the donor block. On GM13, a 2.17-Mb region overlapped *MQTL4* and the donor block. On GM17, a 3.23-Mb region overlapped *MQTL5* and the donor block (Table 2). Therefore, CSSL-182 is an ideal material for secondary population construction.

**TABLE 2:** The information of CSSL-182 and the overlap regions with the *MQTL* of the number of the three-seeded pod.

Code	Block	Segments length	Introgressed	Interval of	Consensus	Physical intervals	Physical intervals	Intersection	Chromosome
	number	of Block (Mbp)	ratio (%)	molecular marker	QTL	of *MQTL* (Mbp)	of block (Mbp)	intervals (Mbp)	
1	25	6.22	0.66	Block1481 ∼ Block1565	*MQTL2*	2.44 ∼ 4.63	0.24 ∼ 6.46	2.19	GM03
2	71	10.69	1.13	Block6225 ∼ Block6245	*MQTL3*	10.02 ∼ 11.20	7.21 ∼ 17.90	1.18	GM12
3	35	5.84	0.62	Block6666 ∼ Block6684	*MQTL4*	20.83 ∼ 26.06	23.89 ∼ 29.73	2.17	GM13
4	31	3.38	0.36	Block8733 ∼ Block8771	*MQTL5*	3.49 ∼ 6.72	3.43 ∼ 6.81	3.23	GM17
Else	157	58.50	6.17						GM02, 06, 19, 20
Total	319	84.63	8.93	4	4	11.83	26.13	8.77	8

### Quantitative Trait Locus Fine Mapping

An F_2_ segregating population consisting of 970 individuals was developed by backcrossing CSSL-182 with “Suinong 14.” In this population, the mean NThSP was 34.39 (8.45–61.26), and the mean proportion of pods with three seeds was 44.63% (17.33–82.25%). The absolute skewness and kurtosis values were less than 1.0, suggesting that the segregation of the NThSP phenotype was normally distributed (Table 3).

**TABLE 3:** Statistical analysis of traits related to the number of the three-seeded pod in three populations.

Population	Trait	Min.	Max.	Mean ± SD	Range	Skewness	Kurtosis
RILs	NThSP	10.48	51.19	26.77 ± 7.87	10.48 ∼ 51.19	0.51	0.42
	RThSP	27.26	65.22	47.25 ± 7.03	27.26 ∼ 65.22	–0.22	0.09
	NPPP	26.50	96.51	56.34 ± 13.13	26.50 ∼ 96.51	0.55	0.53
CSSLs	NThSP	12.32	49.35	22.13 ± 3.64	12.32 ∼ 49.35	0.99	1.06
	RThSP	19.24	75.21	45.22 ± 5.32	19.24 ∼ 75.21	1.22	0.78
	NPPP	24.17	98.32	68.43 ± 8.21	24.17 ∼ 98.32	1.78	1.39
Secondary group	NThSP	6.00	61.00	34.39 ± 2.34	6.00 ∼ 61.00	0.63	0.03
	RThSP	17.33	82.25	44.63 ± 3.55	17.33 ∼ 82.25	0.66	0.21
	NPPP	23.00	86.00	54.66 ± 6.35	23.00 ∼ 86.00	–0.28	0.49
Germplasm resources	NThSP	7.89	38.44	21.65 ± 6.61	7.89 ∼ 38.44	0.51	–0.23
	RThSP	30.99	61.22	46.22 ± 7.35	30.99 ∼ 61.22	–0.06	–0.69
	NPPP	24.56	78.33	46.45 ± 10.40	24.56 ∼ 78.33	0.24	0.08

*NThSP indicates the trait of the number of the three-seeded pod. RThSP indicates the ratio of the number of the three-seeded pod. NPPP indicates the trait of the number of pods per plant. RILs indicate recombinant inbred lines. CSSLs indicate chromosomal segment substitution lines. Min and Max indicate the minimum and maximum values, respectively.*

**FIGURE 3:**
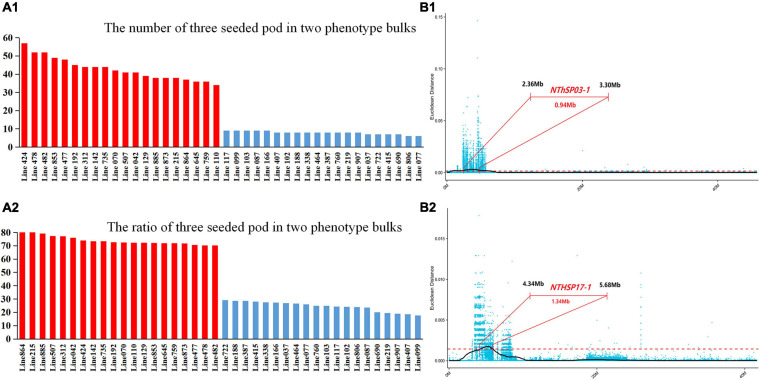
Results of the analysis of two phenotype bulks related to the number of three-seeded pods and quantitative trait locus (QTL) fine mapping. **(A1)** Red and blue bars represent the lines with many and few three-seeded pods, respectively. **(A2)** Red and blue bars represent the lines with a high and low proportion of three-seeded pods, respectively. The two bulks for the bulked segregant analysis included 40 lines. See Supplementary Table 5 for the associated data. **(B1)** Finely mapped QTL for the number of three-seeded pods on chromosome GM03. The QTL interval was 0.94 Mbp long (2.36–3.30 Mbp) and included 42 genes. **(B2)** Finely mapped QTL for the number of three-seeded pods on chromosome GM17. The interval was 1.34 Mbp long (4.34–5.68 Mbp) and included 120 genes.

Two bulks of 20 individuals with extreme phenotypes were selected for BSA to anchor the finely mapped QTL intervals. For the high-NThSP bulk, the NThSP and the proportion of pods with three seeds were 34–57 and 70.27–82.22%, respectively. For the low-NThSP bulk, the NThSP and the proportion of pods with three seeds were 6–9 and 17.9–29.17%, respectively (Supplementary Table 5 and Figure 3A1, A2). Two finely mapped QTL intervals were detected on chromosomes GM03 and GM17. For *NThSP03-1*, compared with the reference sequence of CSSL-182, the SNP index of the high-NThSP bulk was 0.–1.0 and that of the low-NThSP bulk was 0.1–0.4. The physical interval of *NThSP03-1* was 2.36–3.30 Mb (0.94 Mb), which was contained in the *MQTL2* physical interval 2.44–4.63 Mb (2.19 Mb) and included 42 genes. For *NThSP17-1*, compared with the reference sequence of CSSL-182, the SNP index of the high-NThSP bulk was 0.85–1.0, and the low-NThSP bulk was 0.15–0.4. The physical interval of *NThSP17-1* was 4.34–5.68 Mb (1.34 Mb), which was contained in the *MQTL5* physical interval 3.49–6.72 Mb (3.23 Mb) and included 120 genes. Two finely mapped QTL intervals included 162 genes. On chromosome GM03, the candidate genes could be divided into three sections, namely, pollen germination, mRNA modification, and ATP-dependent helicase activity. The genes found on chromosome GM17 could then be divided into different sections depending on the gene function annotation, such as cellulose synthesis, metabolic processes, or other biochemical pathways (Figure 3B1, B2 and Supplementary Table 6).

### Candidate Gene Prediction

**FIGURE 4:**
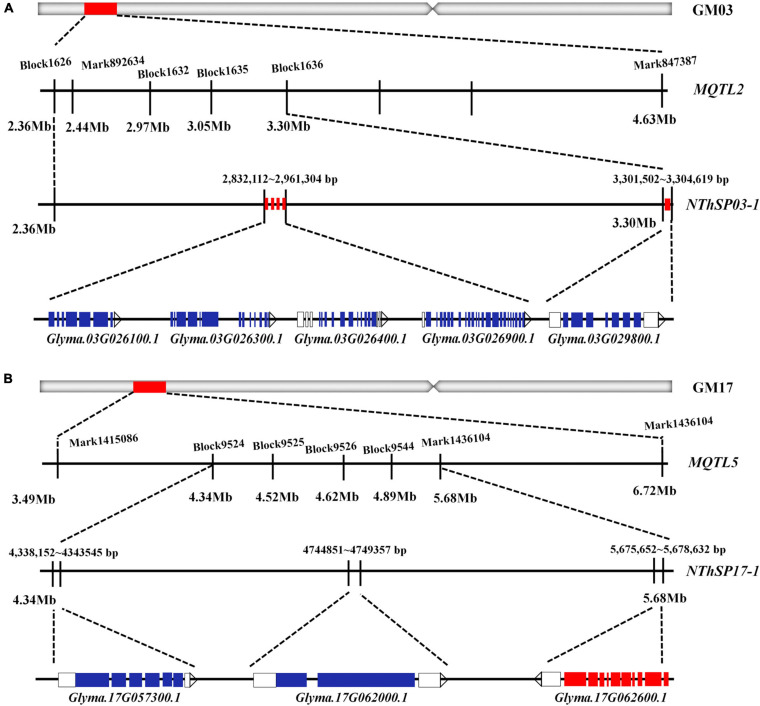
Candidate gene mining on chromosomes GM03 and GM17. **(A)** Consensus QTL, finely mapped QTL, and candidate genes related to the number of three-seeded pods on chromosome GM03. **(B)** Consensus QTL, finely mapped QTL, and candidate genes related to the number of three-seeded pods on chromosome GM17.

Previous research has shown that ovule, pollen, stamen, embryo, and flower development considerably influence pod and seed sets in the soybean (Mukhtar and Coyne, 1981; Tischner et al., 2003; Mena-Alí and Rocha, 2005; Endo and Ohashi, 2009; Kurdyukov et al., 2014; Jiang et al., 2015; Qi et al., 2015). Gene functions related to these biological processes may affect NThSP. Based on this, 8 candidate genes were selected from 162 genes in the target intervals. Homologous gene analysis was performed in the soybean and *Arabidopsis* genomes. For eight genes, five candidate genes were detected in the *NThSP03-1* interval (i.e., *Glyma.03G026100.1*, *Glyma.03G026300.1*, *Glyma.03G026400.1*, *Glyma.03G026900.1*, and *Glyma.03G029800.1*). The homologous genes in the soybean genome were *Glyma.01G140600.1* and *Glyma.17G138200.1* (Zhao et al., 2017; Chen et al., 2018), whereas the homologous genes in the *Arabidopsis* genome were *AT1G71820* and *AT2G45190* (Nolan et al., 2009; Lenser et al., 2016; Pathak et al., 2016). Based on previous studies, functional characterization of these genes indicated that they are involved in pollen germination; pollen tube growth; meristem growth; ovule, embryos, flower, and fruit development; and cell division. Another three candidate genes were included in the *NThSP17-1* interval (*Glyma.17G057300.1*, *Glyma.17G062000.1*, and *Glyma.17G062600.1*). The homologous genes in the soybean genome are *Glyma.13G101900.1*, *Glyma.13G097600.1*, and *Glyma.13G096900.1* (Wang et al., 2015), whereas the homologous genes in the *Arabidopsis* genome are *AT1G73590*, *AT5G57360*, and *AT5G57390* (Bai and Demason, 2008; Kwak et al., 2008; Tsuwamoto et al., 2010; Xue et al., 2012; Hu et al., 2014; Liew et al., 2014; Radoeva and Weijers, 2014; Seefried et al., 2014). Based on previous studies, functional characterization of these genes revealed that they are related to embryo, flower, stamen, embryo development, and seed germination. The information for each gene and its homologous gene from the literature are listed in Table 4. Therefore, these eight genes may be important candidate genes for NThSP (Table 4 and Figure 4).

**TABLE 4:** Candidate genes and their homologous genes in soybean and *Arabidopsis* genome.

Code	Candidate genes	Soybean homologous	References	Arabidopsis homologous	References	Common annotation information of biological process
1	*Glyma.03G026100.1*	*Glyma.01G140600.1*	Chen et al., 2018	*AT1G71820*	Kim et al., 2009; Safavian et al., 2015	Pollen germination, pollen tube growth
2	*Glyma.03G026300.1*	*Glyma.01G140600.1*	Chen et al., 2018	*AT1G71820*	Kim et al., 2009; Safavian et al., 2015	Pollen germination, pollen tube growth
3	*Glyma.03G026400.1*	*Glyma.01G140600.1*	Chen et al., 2018	*AT1G71820*	Kim et al., 2009; Safavian et al., 2015	Pollen germination, pollen tube growth
4	*Glyma.03G026900.1*	*Glyma.01G140600.1*	Chen et al., 2018	*AT1G71820*	Kim et al., 2009; Safavian et al., 2015	Pollen germination, pollen tube growth
5	*Glyma.03G029800.1*	*Glyma.17G138200.1*	Zhao et al., 2017	*AT2G45190*	Lenser et al., 2016; Pathak et al., 2016	Ovule, embryo, flower, fruit development, meristem growth
6	*Glyma.17G057300.1*	*Glyma.13G101900.1*	Wang et al., 2015; Stasko, 2018	*AT1G73590*	Fang, 2008; Hu et al., 2014; Seefried et al., 2014	Embryo development, flower and stamen development
7	*Glyma.17G062000.1*	*Glyma.13G097600.1*	-	*AT5G57360*	Kwak et al., 2008; Xue et al., 2012; Liew et al., 2014	Flower development
8	*Glyma.17G062600.1*	*Glyma.13G096900.1*	-	*AT5G57390*	Tsuwamoto et al., 2010; Radoeva and Weijers, 2014	Positive regulation of embryonic development, ‘seed germination

### Candidate Gene Analysis

Haplotype and qRT-PCR analyses were performed to assess the candidate genes. Of the eight predicted candidate genes, four had different haplotypes in the germplasm resources. The NThSP of the highest line was 38.44 and that of the lowest line was 7.89. The mean NThSP was 21.65 in the germplasm resources. The traits related to the three-seeded pod were approximately normally distributed in the germplasm resources (Table 3). The SNPs of the haplotypes for each candidate gene are listed in Table 5. The NThSP phenotypic differences among the different haplotypes were significant, and four genes were differentially expressed during pod and seed development. The expression levels of the candidate genes reached significant levels during the six developmental periods. The four candidate genes were *Glyma.03G029800.1*, *Glyma.17G057300.1*, *Glyma.17G062000.1*, and *Glyma.17G062600.1* (Tables 5, 6).

**TABLE 5:**
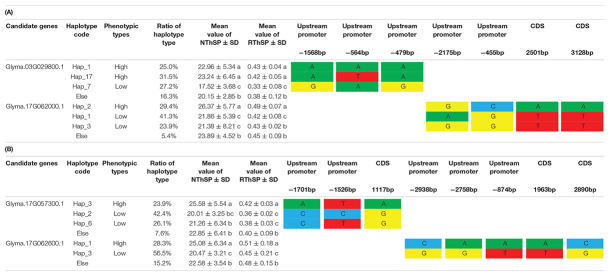
Haplotype analysis of the candidate genes of the number of the three-seeded pod.

*NThSP represents the number of the three-seeded pod, whereas RThSP represents the proportion of the three-seeded pod. The different lowercase letters in columns of each candidate gene indicate the significant difference level (P < 0.05).*

*SNPs are indicated by colored letters.*

**TABLE 6:** Expression analysis of the candidate genes of the number of the three-seeded pod.

Candidate genes	Extremely lines	Phenotypic types	Period and expression indicators					
			R1	R2	R3	R4	R5	R6
Glyma.03G029800.1	Suinong14	CK	5.60 ab	2.70 b	6.77 b	3.39 bc	4.91 ab	5.61 a
	Line215	High	7.60 a	4.65 a	8.55 a	5.93 a	5.32 a	4.36 b
	Line70	High	6.42 a	5.54 a	9.16 a	5.00 a	6.90 a	5.25 a
	Line117	Low	3.49 b	1.93 c	4.96 bc	2.35 c	3.97 c	2.70 c
	Line99	Low	3.93 b	1.18 c	2.27 c	4.61 b	4.10 b	3.29 bc
Glyma.17G057300.1	Suinong14	CK	3.23 b	4.33 a	5.25 a	4.77 ab	4.67 ab	3.92 ab
	Line215	High	5.08 a	5.32 a	6.42 a	5.06 a	5.25 a	5.15 a
	Line70	High	4.04 a	4.60 a	5.42 ab	5.61 a	5.05 a	4.71 a
	Line117	Low	2.68 b	2.32 b	4.42 b	3.06 b	4.25 ab	4.15 a
	Line99	Low	2.04 b	3.02 b	3.73 ab	2.11 c	3.22 b	3.71 b
Glyma.17G062000.1	Suinong14	CK	2.61 ab	2.37 b	4.46 ab	2.34 ab	2.92 ab	2.42 b
	Line215	High	1.44 b	1.18 c	2.60 b	1.77 b	2.43 b	2.30 b
	Line70	High	1.21 b	1.42 c	3.31 b	1.30 b	2.75 b	2.72 b
	Line117	Low	3.91 a	3.68 ab	5.82 a	3.64 a	3.08 a	3.53 a
	Line99	Low	2.91 ab	4.22 a	4.93 a	3.41 a	3.57 a	3.03 ab
Glyma.17G062600.1	Suinong14	CK	4.53 b	3.43 b	5.18 b	3.13 b	4.14 ab	5.61 ab
	Line215	High	6.92 a	5.67 a	7.42 a	6.02 a	5.11 a	6.12 a
	Line70	High	3.52 c	4.32 ab	6.17 a	5.60 a	4.97 a	7.02 a
	Line117	Low	2.55 cd	3.22 b	2.43 c	2.51 c	5.23 a	3.09 c
	Line99	Low	3.04 c	3.95 b	2.90 c	1.96 c	3.55 b	4.30 b

*The different lowercase letters in columns of each candidate gene indicate the significant difference level (P < 0.05).*

For the haplotypes of the four candidate genes, the phenotypes of the high-NThSP lines were significantly higher than the low-NThSP lines in the germplasm resources. In *Glyma.03G029800.1*, the NThSP and proportion of pods with three seeds were significantly higher for Hap-1 (22.96 and 43%) and Hap-17 (23.24 and 42%) than for Hap-7 (17.52 and 33%). The SNPs were located upstream of the promoter at –1,568, –564, and –479 bp. In *Glyma.17G057300.1*, the NThSP and proportion of pods with three seeds were significantly higher for Hap-3 (25.58 and 42%) than for Hap-2 (20.01 and 36%) and Hap-6 (21.26 and 38%). The SNPs were located upstream of the promoter at –1,701 and –1,526 bp and in the CDS region of 1,117 bp. In *Glyma.17G062000.1*, the NThSP and proportion of pods with three seeds were significantly higher for Hap-2 (26.37 and 49%) than for Hap-1 (21.86 and 42%) and Hap-3 (21.38 and 43%). The SNPs were located upstream of the promoter at –2,175 bp and –455 bp and in the CDS region of 2,501 bp and 3,128 bp. In *Glyma.17G062600.1*, the NThSP and proportion of pods with three seeds were significantly higher for Hap-1 (25.08 and 51%) than for Hap-3 (20.47 and 45%). The SNPs were located upstream of the promoter at –2,938 bp, –2,758 bp, and 874 bp and in the CDS region of 1,963 bp and 2,890 bp. The phenotypic differences between the haplotypes for each candidate gene may be the result of these SNP differences (Table 5).

The qRT-PCR analysis indicated that *Glyma.03G029800.1* was more highly expressed in lines 215 and 70 (i.e., the high-NThSP lines) than in lines 117 and 99 (i.e., the low-NThSP lines) or Suinong 14 (recurrent parent) in the R1, R2, R3, R4, and R5 stages. *Glyma.17G057300.1* was more highly expressed in lines 215 and 70 than in lines 117 and 99 or Suinong 14 in the R1, R2, and R4 stages. The expression of *Glyma.17G062000.1* was lower in lines 215 and 70 than in lines 117 and 99 or Suinong 14 in the R1, R2, R3, and R4 stages. *Glyma.17G062600.1* was more highly expressed in line 215 than in Suinong 14 in the R1, R2, R3, and R4 stages. This gene was more highly expressed in line 70 than in Suinong 14 in the R3 and R4 stages. Moreover, the expression level of *Glyma.17G062600.1* was higher in lines 215 and 70 than in lines 117 and 99 in the R3, R4, and R6 stages (Table 6).

## Discussion

The results of this study corroborate those reported in previous studies. For example, the *MQTL1* region on GM02 (42.89–46.19 Mbp) included five QTLs, all of which had negative effects, suggesting that they were stable QTLs throughout the study period. An earlier investigation detected a QTL (*qPN-D1b-2*) using a Satt546 simple sequence repeat marker (physical position: 43,775,564–43,775,623 bp) associated with seed set and seed yield (Ning et al., 2018). Another QTL (*qPN-D1b-3*) detected using Sat_183 (physical position: 44,317,044–44,317,314 bp) and Sat_069 (physical position: 46,353,731–46,353,789 bp) was associated with seed set and seed weight (Ning et al., 2018). A QTL (*QNTPD1b-1*) associated with Sat_135 (physical position: 40,366,215–40,366,272 bp) was reported to be related to the two-seeded pod trait (Asakura et al., 2012; Yang et al., 2013b). Other studies have shown that QTL *qSN-1*, which is associated with Satt350 (physical position: 40,366,215–40,366,272 bp), is also related to the two-seeded pod trait (Asakura et al., 2012; Yang et al., 2013b). Additionally, the QTL *qSN-1*, which is close to the *MQTL1* region on GM02, is associated with Satt189 and Satt350 and is related to early flowering and seed number (Li et al., 2010). Thus, *MQTL1* has been identified by several studies as a genomic locus influencing seed sets. This is an important consensus QTL interval. The *MQTL2* region on GM03 (2.44–4.63 Mbp) includes four QTLs. A previous study revealed that a QTL (*qfn-Chr3*) associated with Satt009 (physical position: 3,910,260–3,910,307 bp) is within the *MQTL2* interval and is related to flower number (Zhang et al., 2010). No loci consistent with *MQTL3* have been reported; thus, this is a new region related to NThSP. The *MQTL4* region on GM13 (20.83–26.06 Mbp) contains a QTL related to the number of ovules per pod; this QTL is associated with Sat_133 (physical position: 23,462,623–23,462,676 bp) (Tischner et al., 2003). Another QTL (*qPN-D2-2*), which is associated with Sat_284 (physical position: 6,551,297–6,551,348 bp) in the *MQTL5* interval on GM17 (3.49–6.72 Mbp), is related to the seed set (Ning et al., 2018). The QTLs identified in earlier studies are associated with flower number, number of ovules per pod, pod, and seed set. These loci are consistent with the consensus QTL identified in this study, which may facilitate the prediction of candidate genes related to NThSP.

Soybean seed yield is positively associated with the number of flowers as well as successful pod and seed set (Egli, 2005; Pushpavalli et al., 2015). The number of seeds per pod is related to the number of ovules that develop in each pod. There are usually five ovules in one flower, but the number of ovules that are aborted ultimately determines the number of seeds in a pod (Shibles et al., 1975; Tischner et al., 2003). Moreover, pollen tube growth, stamen development, and flower development are also important factors (Mukhtar and Coyne, 1981; Tischner et al., 2003; Mena-Alí and Rocha, 2005; Endo and Ohashi, 2009; Kurdyukov et al., 2014; Jiang et al., 2015; Qi et al., 2015; Smitha Ninan et al., 2017). Accordingly, candidate genes with annotated functions related to pollen, stamen, ovule, embryo, and flower development as well as pollen tube growth are likely important for pod and seed set.

In this study, eight genes were annotated with functions associated with pod and seed sets, and homologous genes in the soybean and *Arabidopsis* genomes were functionally annotated. In the *NThSP03-1* interval, four candidate genes (i.e., *Glyma.03G026100.1*, *Glyma.03G026300.1*, *Glyma.03G026400.1*, and *Glyma.03G026900.1*) were identified as homologs in the soybean and *Arabidopsis* genomes. Based on circular RNA differential expression analyses, these genes were found to affect pollen germination and pollen tube growth (Nolan et al., 2009; Safavian et al., 2015; Chen et al., 2018). However, excellent haplotypes for these genes were not detected in the germplasm resources included in this study, implying that these genes are expressed ubiquitously in soybean and are unrelated to seed sets.

The *Glyma.03G029800.1* gene was identified as *GmYABBY5*, which helps maintain meristems during the early floral developmental stages (Zhao et al., 2017). The *Arabidopsis* homolog of this gene is *AT2G45190*, which mediates biological processes related to ovule, embryo, and flower development (Lenser et al., 2016; Pathak et al., 2016). In tomatoes, the *YABBY*-like transcription factor was also found to play a pivotal role in fruit size regulation (Cong et al., 2008). Similar functions of *YABBY5* were also identified in the gynoecium of rice and sugar-apple (Yang and Hwa, 2008). Additionally, this gene underlies the development of ovules (Lora et al., 2011; Cucinotta et al., 2020), supporting that it might also have similar functions to NThSP. In this study, three excellent *Glyma.03G029800.1* haplotypes were detected in the examined germplasm resources. The NThSP data differed significantly among these three haplotypes, indicating that this gene influences the NThSP phenotype. The CAAT-box is a common *cis*-acting promoter element that is recognized by *trans*-acting factors that increase promoter activity. In the *Glyma.03G029800.1* promoter region, a base change from A to G at –1,568 and –479 bp adversely affects the CAAT-box promoter element, which may explain the observed decrease in NThSP. Additionally, the detected base changes in the promoter regions of *Glyma.17G057300.1* and *Glyma.17G062600.1* were also within the CAAT-box element. The different haplotypes of these two genes were associated with similar phenotypic changes. The pod and seed sets may be closely related to increased gene expression resulting from a functional CAAT-box element. The qRT-PCR analysis provided additional information regarding the potential gene functions. High *Glyma.03G029800.1*, *Glyma.17G057300.1*, and *Glyma.17G062600.1* expression levels may promote pod and seed sets. There were significant differences in the expression levels of these genes between the lines with a high NThSP and those with a low NThSP.

The *Glyma.17G057300.1* gene was identified as *GmPIN2b*. A previous study confirmed that upregulated *GmPIN2b* expression can induce soybean flower growth and grain development (Wang et al., 2015). The *AT1G73590* gene is the *Arabidopsis* homolog of *PIN1* and *Glyma.17G057300.1*. This gene mediates *Arabidopsis*, tomato, and soybean flower and fruit development. Earlier research confirmed that *AT1G73590* expression affects auxin transport in embryos (Bai and Demason, 2008; Hu et al., 2014; Seefried et al., 2014). Accordingly, *Glyma.17G057300.1* may be another candidate gene related to NThSP. In this study, different *Glyma.17G057300.1* haplotypes were detected in the analyzed germplasm resources, with very significant differences in the phenotypes associated with the haplotypes. In the CDS of this gene, the A base at 1,117 bp resulted in an aspartic acid in the encoded protein as well as a high NThSP, whereas a G at this position resulted in glycine and a low NThSP. The base change in *Glyma.17G062600.1* CDS (2,890 bp) similarly affected NThSP. More specifically, a C at this position resulted in alanine and a high NThSP, whereas a G at this position resulted in glycine and a low NThSP. The qRT-PCR data may be useful for functionally characterizing *Glyma.17G057300.1* and *Glyma.17G062600.1.*

In this study, a RIL was used to identify QTL potentially related to NThSP. A CSSL was used to verify the candidate QTL intervals. A secondary group was used to map the fine QTL. Another germplasm resource population was used to check the candidate genes by haplotype identification. Comparing four populations, the range of NThSP and three-seeded trait values in the RILs, CSSLs, and secondary group was greater than in the germplasm resources. This indicated evident trait separation in the targeted populations. However, the haplotype analysis of candidate genes in the germplasm resources was more objective. The haplotypes and NThSP phenotype could be better validated in the germplasm resources. In this study, most of the higher NThSP phenotype lines, including the highest one, had the same haplotype. For example, there were 27 lines, accounting for 29.35% of the germplasm resources, with a higher NThSP phenotype that had the same haplotype (Table 5). This suggested that the candidate gene haplotype was probably related to the NThSP phenotype. However, it is possible that there were few higher NThPP lines that did not share the same haplotype with it. These lines with different haplotypes and higher NThPP phenotypes belonged to the else Haps, which could be attributed to other genes. It is likely that other NThSP genes exist in the study population. Additionally, CSSL-182, which included the genomic segments imported from the donor, had a better phenotype than the recurrent parent. This was conducive to the segregation and recombination of the target intervals in the secondary group and the fine mapping of NThSP QTL. Candidate genes were selected strictly according to functional annotations. Thus, there may be other candidate genes related to NThSP in the QTL intervals. The selected candidate genes were annotated with functions related to pollen tube growth, as well as the development of pollen grains, stamens, ovules, embryos, and flowers. An analysis of the candidate genes revealed that they are closely related to pods and seed sets. Future studies should more precisely characterize the functions of the identified candidate genes in transgenic soybean plants. In addition, there were different haplotypes in the germplasm resources for the candidate genes. Several SNPs existed in the different haplotypes. These SNPs could be developed into KASP (competitive allele-specific polymerase chain reaction) markers, and the KASP markers could be evaluated in the segregated populations to confirm their function for the selection of high-NThSP materials in molecular marker-assisted breeding.

## Data Availability Statement

The datasets presented in this study can be found in online repositories. The names of the repository/repositories and accession number(s) can be found in the article/Supplementary Material.

## Author Contributions

QC and FL: conceptualization. ZQ and HJ: methodology. DX, HJ, and XW: validation. CDL, TG, ZW, CYL, and NW: formal analysis. WZ, HZ, ZYZ, RZ, and DS: data curation. CDL: writing-original draft. DX, HJ, and QC: writing-review and editing. ZH: project administration. All authors have read and agreed to the published version of the manuscript.

## Conflict of Interest

The authors declare that the research was conducted in the absence of any commercial or financial relationships that could be construed as a potential conflict of interest.

## Publisher’s Note

All claims expressed in this article are solely those of the authors and do not necessarily represent those of their affiliated organizations, or those of the publisher, the editors and the reviewers. Any product that may be evaluated in this article, or claim that may be made by its manufacturer, is not guaranteed or endorsed by the publisher.

## Footnotes

1
http://www.soybase.org

2
http://www.ncbi.nlm.nih.gov

3
www.genome.jp/kegg/pathway.html

4
www.geneontology.org/

5
https://phytozome.jgi.doe.gov/pz/portal.html

6
https://haploview.software.informer.com

7
http://www.ub.edu/dnasp/
